# Effect of Tea Polyphenols on the Storage Stability of Non-Fermented Frozen Dough: Protein Structures and State of Water

**DOI:** 10.3390/foods12010080

**Published:** 2022-12-23

**Authors:** Kai Zheng, Zhehan Chen, Yang Fu, Lei Chen, Xiangwei Zhu, Xi Chen, Wenping Ding

**Affiliations:** 1 School of Food Science and Engineering, Wuhan Polytechnic University, Wuhan 430023, China; 2 Key Laboratory for Deep Processing of Major Grain and Oil, Ministry of Education, Hubei Key Laboratory for Processing and Transformation of Agricultural Products, Wuhan Polytechnic University, Wuhan 430023, China; 3 National “111” Center for Cellular Regulation and Molecular Pharmaceutics, Key Laboratory of Fermentation Engineering (Ministry of Education), Hubei Key Laboratory of Industrial Microbiology, Hubei University of Technology, Wuhan 430068, China

**Keywords:** tea polyphenols, frozen wheat dough, water distribution, secondary protein structure

## Abstract

The usage of tea polyphenols (TPs) as a natural food additive into non-fermented frozen dough (NFFD) has rarely been investigated, and results have been controversial. Hence, this study investigated the effect of TPs at various levels (0, 0.5, 1, and 2%) on the quality of NFFD stored from 0 to 4 weeks. The rheological characteristics, water state, protein, and its microstructure were analyzed by DSC, LF-NMR, SDS-PAGE, FT-IR, and SEM, respectively. Results showed that the 0.5% TP group delayed the deterioration of protein and inhibited the water migration in dough throughout the whole frozen storage period. In addition, the 0.5% TP group enhanced the rheological properties of NFFD and stabilized the sulfhydryl content and the secondary structure in the gluten network. On the contrary, opposite phenomena were found in the 1 and 2% TP groups, which might be due to the induction of excess hydroxyl groups from TPs. In conclusion, our results suggested that a proper addition of TPs, but not an excessive amount (>1%), exhibited beneficial effects in maintaining the quality of NFFD during the 4-week frozen storage. Moreover, this paper elucidated the mechanism of TPs in influencing the protein structure and water state of NFFD during storage and provided new insight into its application in dough-based foods.

## 1. Introduction

Frozen dough has been widely used as a promising technology for standardizing product quality and saving costs in dough products during the past few decades [1]. However, the integrity of the gluten network in the dough could be affected by the ice crystals formed during freezing and their re-crystallization caused by temperature change during storage [2]. A possible explanation for the deterioration would be the disruption of disulfide links of gluten, which leads to the depolymerization of high-molecular-weight gluten proteins [3]. A promising strategy to deal with this issue is to add proper food additives. Tea polyphenols (TPs) naturally exist in tea leaves and possess several functional properties [4]. Numerous studies have demonstrated that phenolics could interact with dough ingredients such as water, gluten proteins, and starch to change the physicochemical properties of the dough [5,6,7]. For example, Rui Liu et al. [8] reported that the adding of oligomeric proanthocyanins could enhance the quality of dough by compacting the gluten protein network. Meanwhile Wang et al. [9] demonstrated that tannins could modify wheat flour by combining their hydroxyl groups with the free amino groups of the gluten and non-gluten proteins.

In the last decade, investigations on adding TPs to fermented frozen dough have received substantial attention by scientists. It was reported that the volume and hardness of fermented frozen dough were either reduced [10] or increased [11] by TPs due to different mechanisms. However, studies regarding the effect of TPs on NFFD were rarely investigated, and results were controversial. It was reported that TPs enhanced the stability of the dough and promoted the formation of gluten networks [12], while another study [11] showed that TPs hindered the development of gluten networks by breaking disulfide bonds, which led to a poor dough quality. Thus, more studies should focus on this area to draw a conclusion on how TPs influence the NFFD’s quality and explain the underlying mechanisms. Furthermore, most traditional Chinese dough products are made from non-fermented dough, particularly dumplings and noodles, which are widely consumed in northern China [13]. There is an urge to meet the increasing demand of NFFD products as the population continuously grows and the pace of work life accelerates. 

Therefore, the aims of this work were to investigate the effect of TPs on the water state and protein structure of NFFD. The NFFD was prepared by adding TPs at different levels (0–2%), and the rheological characteristics, molecular weight distribution of protein, sulfhydryl content, protein secondary structure, frozen water content, and microstructure of the NFFD from 0 to 4 weeks were all carefully examined. This study would advance the use of TPs in NFFD and offer fresh insights into their transformation into effective dough-freezing agents.

## 2. Materials and Methods

### 2.1. Materials

A commercial medium-gluten wheat flour was purchased from Yihai Kerry Arowana Cereals, Oils & Foodstuffs Co., Ltd. (Zhoukou, China). The ash, moisture, wet gluten, and crude protein were measured to be 0.42%, 12.79%,11.75%, and 37.56%, respectively (calculated on a wet basis). TPs (mainly composed of ECG, EGC, EGCG, EC, etc.) were purchased from Shanghai Yuanye Bio-Technology Co., Ltd. (Shanghai, China). The edible salt was purchased from Hubei Salt Group, Ltd. (Xiaogan, China). All chemicals and reagents were of at least analytical grade. Ultrapure water with a resistivity of 18.2 MΩ·cm was used in all experiments.

### 2.2. Preparations of the Dough

**Figure 1:**
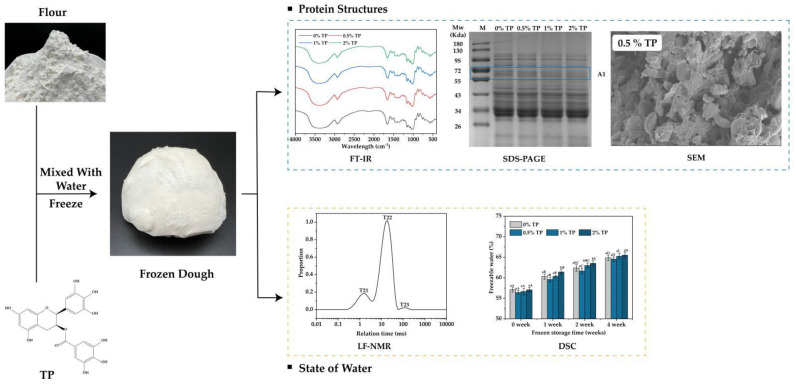
Scheme of experimental design. The protein structure was analyzed by FT−IR, SDS−PAGE, SEM, etc., and the water state was analyzed by LF−NMR and DSC. TP: Tea Polyphenol.

Briefly, 500 g wheat flour, 225 g ultrapure water (20–25 °C), and 5 g edible salt were mixed and kneaded at a low speed (4 min) and then a top speed (8 min) in a vertical two-speed dough mixer (H20F, Guangdong Lifeng Machinery Manufacturing Co., Ltd., Shenzhen, China) until the gluten network formed. The mixed dough was then allowed to stand for 30 min. The addition levels of TPs were 0% (control), 0.5%, 1%, and 2% of the flour weight. The dough was cut evenly into small pieces of 60 g each. After that, they were wrapped in polyethylene bags and stored at −18 °C for 0 to 4 weeks. The NFFD was then taken out at different storage times for analyzing the change in protein and water state; the scheme of the following works are shown in Figure 1.

### 2.3. Rheology Tests

The thawed NFFD samples’ rheological properties at 0–4 weeks were characterized by a DHR-2 controlled stress rheometer (TA instruments, New Castle, DE, USA). The dough was frozen for 1.5 h and then defrosted at 25 °C for 1.5 h before testing (the samples from week 0 were examined after being quickly frozen and then thawed). A piece of dough was loaded between the two 25 mm parallel plates at a 0.5 mm gap. The excess edges of samples were cut and then conditioned for 5 min. A frequency sweep test at a range of 0.1 to 10 Hz was performed to evaluate the rheological variations of each sample [14]. All tests were at 25 °C, with a fixed strain of 0.5% within the linear viscoelastic region. The storage modulus (G′) and loss modulus (G″) were then calculated at a 1 Hz frequency.

### 2.4. SDS-PAGE 

SDS-PAGE analyses were conducted with the separation gel (10%) and concentrated gel (5%) as described by Zhang et al. [15] Briefly, the NFFD at both 0 and 4 weeks was freeze-dried and ground into powder, 50 mg of which were mixed with a buffer containing 0.125 mol/L Tris-HCl, 2 mg/mL SDS, 20 mg/mL glycerol, and 0.01 mg/mL Bromophenol Blue. The mixture was then reacted for 3 h at room temperature and centrifuged at 10,000× *g* (4 °C, 20 min). After that, the supernatant was put into a 100 °C water bath for 5 min and then loaded on the electrophoresis gel at a fixed 15 mA current. The voltages of concentrated and separation gel were 80 V and 100 V, respectively. The gels were stained with 1 g/L Coomassie Brilliant Blue R-250 and then decolorized in a solution consisting of 800 mL distilled water, 100 mL glacial acetic acid, and 100 mL methanol. Each experiment was independently carried out three times.

### 2.5. Determination of Free Sulfhydryl Groups 

The free SH content was determined as previously reported [16]. Briefly, 50 mg of the prepared dough powder were dispersed in 1 mL of the 0.2 M Tris-Gly buffer (pH 8.0, 8 M urea, and 3 mM 5,5′ -Dithiobis-2-nitrobenzoic acid) and diluted to 10 mL with the buffer after adding 4.7 g of guanidine hydrochloride to make the reaction solution.

An aliquot of the solution (1 mL) was mixed with 50 uL Ellman’s reagent, 4 mL 8 M urea, and 5 M guanidine hydro-chloride solution, and the mixture was allowed to stand at room temperature for 20 min. The absorbance was measured at 412 nm, and the calibration curve with reduced glutathione ranging from 0–1 mM/L was used to convert it into the content of free sulfhydryl groups. Each determination was measured three times.

### 2.6. FT-IR

A Nicolet iS5 FTIR instrument (Thermo, Waltham, MA, USA) was used to characterize the secondary structures of protein in the NFFD at both 0 and 4 weeks, at a resolution of 4 cm^−1^ [17]. The freeze-dried NFFD was ground into powder, thoroughly mixed with KBr (1:100 *w*/*w*), and pressed into thin slices, then scanned at a range of 4000–400 cm^−1^ with a total of 32 scans. The baseline correction of amide I band (1600–1700 cm^−1^) was performed with both OMNIC and Peakfit 4.0 software, and the curve was fitted by Gaussian smooth deconvolution to calculate the distribution of secondary structures of the protein.

### 2.7. Freezable Water Content

The freezable water content in NFFD was investigated by a Q2000DSC (TA, USA), according to Xin and coworkers [18], with slight modifications. NFFD samples at 0–4 weeks were thawed at room temperature, weighed approximately 15 mg, and sealed in an aluminum DSC pan. The NFFD sample was then cooled to −30 °C with liquid nitrogen, equilibrated at −30 °C for 5 min, and scanned at a heating rate of 5 °C/min from −30 °C to 30 °C. The moisture content and enthalpy of each sample were determined in triplicate. The freezable water content (*C_FW_*) in NFFD was calculated as follows:

(1) $$C_{FW}=\frac{\Delta Hm}{\Delta Hi\text{×}W_{A}}$$

where *C_FW_* is the content (%, wet basis) of freezable water in NFFD, Δ*Hm* is the melting enthalpy, Δ*Hi* is the fusion enthalpy of ice (334 J/g), and *W_A_* is the moisture content (%, wet basis).

### 2.8. LF-NMR

Proton relaxation analyses were performed by NMI20-040 V-1 NMR spectrometer (Niu Mai Electronic Technology Co., Ltd., Suzhou, China) according to Lu et al. [19]. T_2_ relaxation time was analyzed at a ^1^H resonance frequency of 20 MHz. A slice (5.0 ± 0.01 g) from the NFFD was cut and put into an NMR test tube. Then, the tube was placed in the center of a radio frequency coil in the permanent magnetic field at 32 °C. T_2_ was measured by parameters set for the Carr–Purcell–Meiboom–Gill (CPMG) sequence as follows: successive scans = 3000 ms, echo number = 2000, echo time = 0.2 ms, and scan number = 8. Each NFFD sample was measured three times. Carr–Purcell–Meiboom–Gill (CPMG) pulse sequences were fitted by T_2_-fit program.

### 2.9. SEM

An S-3000 N SEM instrument (Hitachi, Tokyo, Japan) was used to characterize the microstructures of NFFD samples at both the 0th and 4th week, with some modifications according to Tang et al. [20]. The NFFD samples were freeze-dried then cut into small slides (0.5 cm × 0.5 cm × 0.5 cm). They were then coated with gold sputtering and observed at 2000 times amplification with 10 KV accelerating voltage.

### 2.10. Statistical Analysis

All experiments were conducted three times, and the data were analyzed by one-way ANOVA using Duncan’s test using the software SPSS 19 (SPSS Inc., Chicago, IL, USA). Data were expressed as mean ± standard deviation at a significance level of *p* < 0.05.

## 3. Results and Discussion

### 3.1. Effect of TP on the Rheological Behavior of NFFD

**Figure 2:**
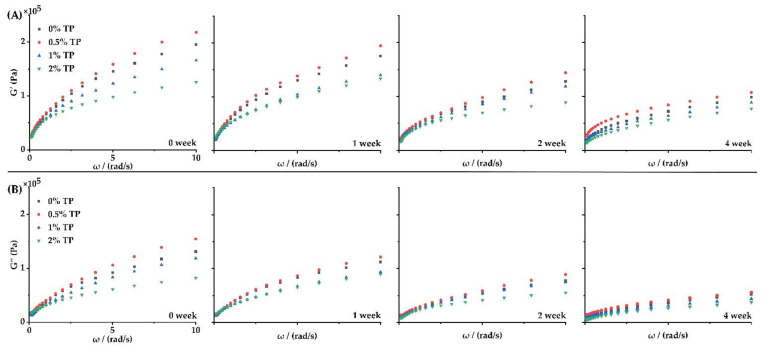
Effect of TPs on G′ (**A**) and G″ (**B**) of the NFFD (0.1–10 Hz frequency, 25 °C) at 0–4 weeks.

To study the rheological behavior of dough with TPs during frozen storage, the storage modulus (G′) and loss modulus (G″) of the dough were determined at 0–4 weeks. As shown in Figure 2, the dough samples showed typical solid viscoelastic rheological behavior at all weeks, as the values of G′ were generally higher than that of G″. The values of G′ and G″ for the 0.5% TP group were the highest among all groups during the whole storage duration, while the G′ and G″ values decreased in the 1 and 2% TP groups in a dose-dependent manner. These results indicated that 0.5% TP contributed to the polymerization of wheat gluten and enhanced the gluten strength of the dough, which was in accordance with the findings of Pan et al. [10]. TPs would help to increase the dough’s toughness and elasticity, as well as its tenderness when in an appropriate quantity [21]. However, higher levels of TPs (1–2%) would lead to an opposite phenomenon, since excessive TPs in the dough system not only dilute the gluten protein network, but also introduce a significant amount of hydroxyl groups, which reduces the hydrogen bonding connection between starch chains and inhibits their recrystallization and retrogradation [22]. 

### 3.2. Effect of TP on Protein Bands in NFFD

**Figure 3:**
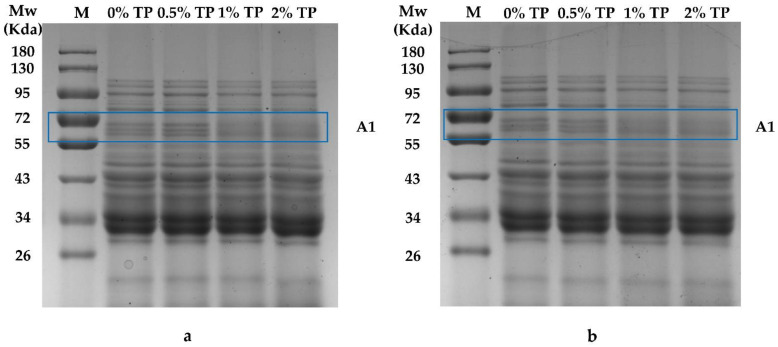
SDS-PAGE patterns of NFFD with 0–2% TP at 0 (**a**) and 4 (**b**) weeks. M, molecular weight of standard; A1, Area 1.

The SDS-PAGE patterns of NFFD containing 0–2% TP at both 0 and 4 weeks are shown in Figure 3. As presented, the protein in the NFFD did not become visible or disappear during the frozen storage. The intensity of protein bands at around 72 kDa (Area 1) was increased when 0.5% TP was added compared with the control (0%); however, it was weakened when more TPs were added at both the 0th and 4th weeks. This phenomenon might be attributed to the epigallocatechin-3-gallate in TPs that is capable of combining with gluten, thus stabilizing the protein structure [21]. This result was consistent with previous findings that TPs revealed a modest increase in protein at around 72 kDa at low concentrations [23], while high levels of TPs would lead to the exchange of the sulfhydryl/disulfide bond in the gluten network and gradually cause depolymerization of the gluten, which would degrade into lower-molecular-weight proteins [24], such as α, β-gliadins (26–43 KDa) [25]. In addition, the protein band intensity at 55–72 kDa (ω-gliadins) decreased as the frozen storage duration increased. The depolymerization of high-molecular-weight proteins might be due to the destruction of covalent and non-covalent interactions between gliadin and glutenin polymers [26]. The mechanical force that is created by the formation of ice crystals in the NFFD would be another reason for the depolymerization, since the mechanical force could break down the gluten network [27]. Moreover, the study also showed that TPs could increase the stability of ω-gliadins [23], which is consistent with our present study. 

### 3.3. Effects of TPs on Free Sulfhydryl Contents 

**Figure 4:**
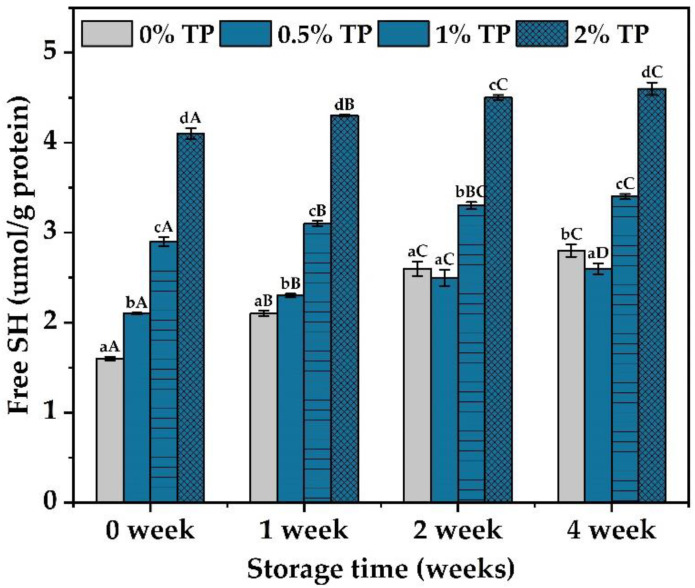
Sulfhydryl contents of gluten at 0 to 4 weeks, stored at −20 °C with various TP additions. Each experiment was carried out in triplicate. Values with different lower- or uppercase letters differ significantly among samples, either with the same frozen storage time or TP addition (*p* < 0.05).

Free sulfhydryl groups could influence the elongation, adhesion, and swelling of the gluten network, thus change the physicochemical properties of the dough [16]. Therefore, the free sulfhydryl contents were characterized in the NFFD at 0–4 weeks. Results showed that the free sulfhydryl content was slightly decreased at the 3rd week and significantly decreased at the 4th week in the 0.5% TP group when compared with the control (0% TP) (Figure 4), which indicated the protection of the disulfide bond by TPs within the gluten networks. The ability of TPs to enhance the hydrogen bond, hydrophobic interaction, and water–solid interaction within the gluten network would lead to increased strength of the gluten and thus reduce the breakdown of the disulfide bond [23]. However, such protection against protein deterioration did not continue with the increase in TP contents, since an opposite trend of free sulfhydryl content was observed when more TPs were added (Figure 4, 1–2% TP groups). As discussed in Section 3.1, the introduction of hydroxyl groups by TP [22] could hinder and reduce the formation of disulfide bonds through the redox reaction [28], leading to the increase in free sulfhydryl content.

### 3.4. Effects of TPs on Protein Secondary Structures

**Figure 5:**
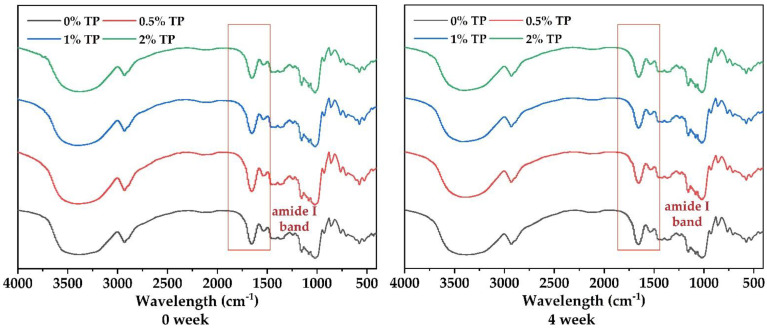
Infrared spectra of NFFD at both 0 and 4 weeks, stored at −20 °C with various TP additions.

To further investigate the effect of TPs on protein structure in the NFFD, the FTIR spectra of dough samples with 0%, 0.5%, 1%, and 2% TP at both 0 and 4 weeks were recorded (Figure 5). In the meantime, the proportions of protein secondary structure were calculated through Peakfit based on the absorption peaks in the 1650–1660 cm^−1^ (α-helix), 1612–1640 cm^−1^ (β-sheet), 1662–1670 cm^−1^ (β-turn), and 1642–1648 cm^−1^ (irregular curl) regions. 

Amide I band (1600–1700 cm^−1^) is one of the most important characteristic bands to determine protein secondary structure [29]. The characteristic peaks at 1670 cm^−1^ and 1633 cm^−1^ show that the interaction of hydrogen bonds in protein molecules leads to β-turn from the glutamine side chain and β-sheet (Figure 5).

The secondary protein structures were gradually transformed from ordered to disordered when frozen time increased; however, the deterioration could be slightly hindered by 0.5% TP (Table 1). Different TP addition exhibited significant influence on the protein secondary structures. The proportion of β-sheet was slightly increased in the 0.5% TP group and then significantly decreased in the 1 and 2% groups at 0and 4 weeks, respectively (Table 1), suggesting the protection effect of TPs in proper amounts for the protein structure, as proved by our results in Section 3.3. The protection effect would disappear when 1 and 2% TP were added. Similar behavior was found in α-helix as presented in Table 1. The formation of α-helix and β-sheet in the spatial structure of proteins could be promoted by disulfide bonds [12] and intermolecular hydrogen bonds [23], which were elevated by 0.5% TP and resulted in the stabilizing of the “grid” protein structure. However, when excess amounts of phenols diffused into the “grid” structure, the hydrogen bond would be largely broken, leading to the reduced content of α-helix and β-sheet [9]. As for the β-turn, whose proportion behaved opposite to β-sheet when different levels of TPs were added, this was due to their transformation with each other [28].

### 3.5. Effect of TPs on the Content of Freezable Water of NFFD

**Figure 6:**
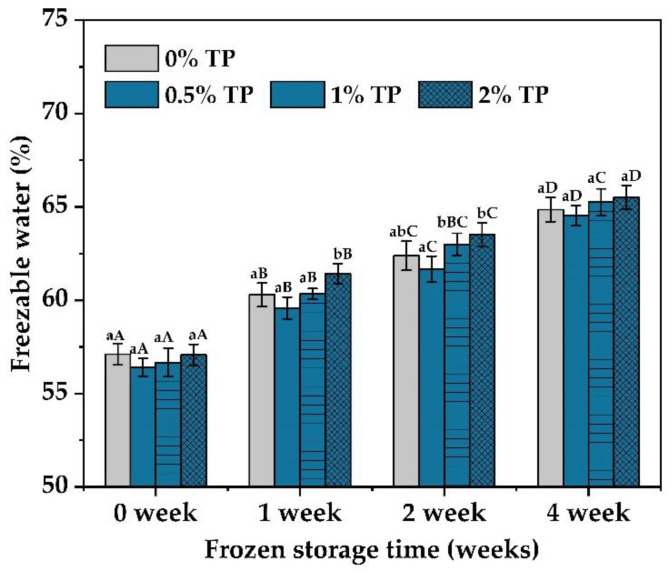
The freezable water content of NFFD (%, wet basis) in the presence of TPs (0–2%) at 0–4 weeks. Values with different lower- or uppercase letters differ significantly among samples either with the same frozen storage time or TP addition (*p* < 0.05).

Freezable water is a key factor affecting the quality of NFFD because of its ability to disrupt the protein network when ice crystals are formed [30]. The amounts of freezable water in the NFFD with different levels of TPs (0–2%) at various storage periods were characterized by DSC. According to Figure 6, the freezable water contents in dough samples increased over time from 0 to 4 weeks. This phenomenon was in line with several other studies [23,31,32] and could be attributed to the development, expansion, and recrystallization of ice crystals in the dough. Those ice crystals further cause the breakdown of the gluten network, along with weaker intermolecular connections, which in turn leads to the release of water molecules and consequently increases the amount of freezable water [33].

In addition, the freezable water content of samples during various frozen times was decreased in the presence of 0.5% TP when compared with the control and then increased as the level of TPs increased. The introduction of hydroxyl groups from TPs (0.5% TP) strengthened the connection between starch and protein molecules and enhanced the gluten network [9]. Thus, more water molecules would be trapped in the gluten protein network, leading to the decrease in the freezable water content. This is in accordance with Wu et al. [12], and these results again confirmed our deduction that the addition of moderate amounts of TPs would promote the water-holding capacity of the NFFD, thus reducing the amount of frozen water. However, the excess increase in TP content (1–2%) would dilute the gluten protein network and result in an opposite phenomenon in the water-holding capacity of the dough [10].

### 3.6. Effect of TPs on the Water State of NFFD

**Figure 7:**
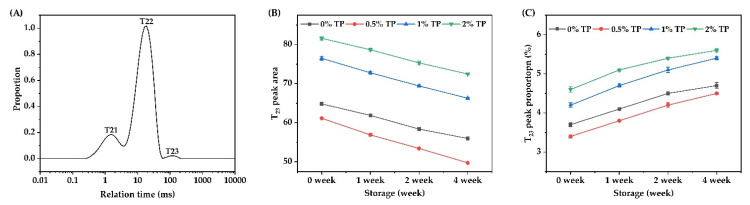
The LF-NMR spin relaxation (T_2_) test of NFFD during storage. (**A**) A typical T_2_ relaxation distribution curve, (**B**) peak area of T_23_, and (**C**) peak area proportions of T_23_.

The water state and its distribution in the NFFD with TPs (0–2%) from 0–4 weeks were analyzed by using LF-NMR, and the results are shown in Figure 7. A typical T_2_ (transverse relaxation time) curve of NFFD was shown in Figure 7A, within which three proton populations, T_21_ (0.01–4 ms), T_22_ (4–75 ms), and T_23_ (75–205 ms), are represented for the bound water, immobilized water, and free water, respectively [32].

To further elucidate the changes in free water distribution in the NFFD, the corresponding peak area and proportion of T_23_ are shown in Figure 7B,C. The peak area and proportion of the 0.5% TP group were the lowest among all the groups throughout the whole frozen duration from 0 to 4 weeks, indicating that the content of free water was the lowest in the 0.5% TP group over time. The slight inclusion of TPs introduced a small amount of hydroxyl groups, which contributed to the binding of water molecules on the surface or within the structure of the gluten network during dough formation, allowing full absorption of water and maintaining the dough quality over the course of storage [8]. The peak area and proportion (Figure 7B,C) were increased by adding more TPs (1–2%), since more hydroxyl groups would competitively bind with water molecules to the gluten network due to the hydrophilic nature of TPs, reducing the water absorption of the dough and therefore affecting dough quality [34].

### 3.7. Effect of TPs on the Microstructures of NFFD

**Figure 8:**
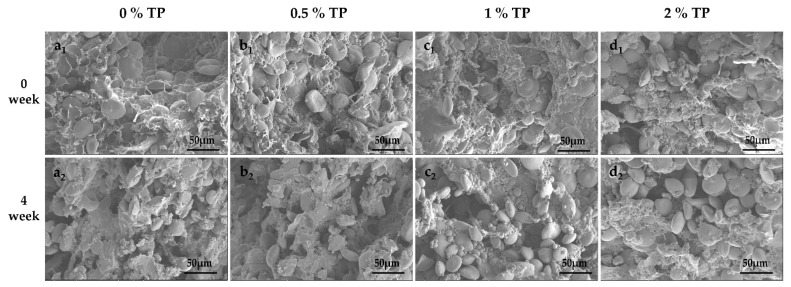
Microstructures of NFFD with TPs at different storage periods (**a**–**d** represent 0% TP, 0.5% TP, 1% TP, and 2% TP, respectively).

The microstructures of NFFD with 0–2% TP at both 0 and 4 weeks were visualized by SEM; the results are presented in Figure 8. In general, the starch granules were embedded in the gluten network, forming a laminar structure. As the storage period increased, the gluten network was affected, and the starch granules were exposed regardless of TP addition. This is because the size of ice crystals gradually increased over time, leading to the disruption of the gluten structure [32]. A similar phenomenon was reported by Jiang et al. [35] and Wang et al. [36].

The fresh dough (0 weeks) without TPs showed a continuous and compact gluten network with starch granules embedded (Figure 8a_1_). By adding TPs (0.5%), the gluten network structure became denser and more ordered, leading to a more aggregated morphology of starch granules (Figure 8b_1_). The density of the gluten network could be caused by the interaction between TPs and gluten [37], which further resulted in the aggregation of starch granules. In addition, the hydrophilic nature of TPs might be another reason contributing to the tight binding to the gluten network [12].

As presented, the gluten network was partially broken down and more starch granules gradually exposed when 1% and 2% TP were added (Figure 8c_1–2_,d_1–2_). This might be due to the fact that TPs have a polyhydroxy structure, so excessive addition of TPs will reduce the disulfide bond in the dough and affect the stability of the gluten network in the dough [38]. A porous structure was observed in dough samples stored up to 4 weeks with 0–2% TP. However, the 0.5% TP group showed the tiniest pores compared with other groups at the 4th week. This may be attributed to the binding of TPs to gluten proteins to form a tea polyphenol–gluten protein cross-linked meshwork structure. The research results of Chen et al. [39] also showed that the phenolic hydroxyl group of TPs would combine with the peptide group on the main chain of the protein and -OH, -NH_2_, and -COOH on the side chain in the way of a hydrogen bond at multiple points, thus stabilizing the protein network structure. Nevertheless, the addition of excessive TPs not only diluted the gluten protein network, but also reduced the disulfide bonds in the dough and destroyed the structure of the gluten protein network in the dough, resulting in the exposure of starch. This result is consistent with the results of SDS-PAGE and changes in free sulfhydryl groups.

## 4. Conclusions

Our study evaluated the effect of TPs at different addition levels (0–2%) on the protein structure and water state of NFFD from 0 to 4 weeks. In summary, the inclusion of 0.5% TP exhibited a beneficial effect on maintaining the dough’s quality. Those beneficial effects include increasing the dough’s toughness, restraining the increase in the freezable water content of the dough, and delaying the deterioration of protein during frozen storage. An adequate amount of hydroxyl groups introduced by TPs would promote the gluten network of NFFD by forming intermolecular hydrogen bonds with gluten to stabilize the “grid” structure and mitigate the damage of ice growth and recrystallization of NFFD. However, the addition of higher amounts of TPs would cause the exchange of sulfhydryl/disulfide bonds in the gluten network and gradually depolymerize the gluten into smaller-molecular-weight proteins, leading to the deterioration of the gluten network. This study would advance the use of TPs in NFFD and offers fresh insights into their transformation into effective dough-freezing agents. To further explain the underlying mechanisms of different TPs such as ECG, EGC, and EGCG, our future work will be focused on the effect of a pure TP compound on the quality of NFFD during frozen storage.

## Figures and Tables

**Table 1:** The effect of TP addition on protein secondary structures in NFFD (*n* = 3).

Time/Weeks	Addition (TP)/%	Secondary Protein Structure			
		β-Sheet/%	Irregular Curl/%	α-Helix/%	β-Turn/%
0	0	30.02 ± 0.13 c	16.61 ± 0.06 b	37.44 ± 0.08 c	15.93 ± 0.12 b
	0.5	30.19 ± 0.04 c	16.23 ± 0.13 a	38.72 ± 0.06 d	14.86 ± 0.07 a
	1	28.78 ± 0.08 b	16.50 ± 0.09 b	36.71 ± 0.07 b	18.01 ± 0.14 c
	2	27.82 ± 0.11 a	18.39 ± 0.14 c	35.72 ± 0.11 a	18.07 ± 0.21 c
4	0	29.65 ± 0.14 c	16.93 ± 0.11 b	37.02 ± 0.15 c	16.40 ± 0.25 b
	0.5	29.81 ± 0.08 c	16.56 ± 0.09 a	38.55 ± 0.16 d	15.08 ± 0.11 a
	1	27.61 ± 0.13 b	17.12 ± 0.17 b	36.08 ± 0.08 b	19.19 ± 0.23 c
	2	26.59 ± 0.21 a	18.88 ± 0.22 c	35.32 ± 0.21 a	19.21 ± 0.26 c

Values with different letters indicate significant difference among samples at the same frozen storage time (*p* < 0.05).

## Data Availability

The data are contained within the article.
